# Effect of prior diverse surgical histories on the efficacy and safety of one-stage retrograde intrarenal surgery in patients with renal stone: a retrospective study

**DOI:** 10.3389/fruro.2026.1735018

**Published:** 2026-03-10

**Authors:** Daocheng Fang, Dong Mingyuan, Shuangquan Sun, Hui Wang, Hui Wen

**Affiliations:** Department of Urology, Songjiang Hospital Affiliated to Shanghai Jiaotong University School of Medicine, Shanghai, China

**Keywords:** intrarenal surgery, percutaneous nephrolithotomy, renal stones, retrograde intrarenal surgery, surgical history

## Abstract

**Purpose:**

To investigate the influence of a history of percutaneous nephrolithotomy (PCNL) and a history of retrograde intrarenal surgery (RIRS) on the efficacy and safety of one-stage RIRS in patients with renal stones.

**Methods:**

A retrospective analysis was conducted on the clinical data of 115 patients with renal stones who underwent one-stage RIRS from January 2022 to June 2025. These patients were divided into three groups: group A (with a history of PCNL) comprising 28 cases, group B (with a history of RIRS) comprising 37 cases, and group C (without a history of urinary stone surgery) comprising 50 cases. The operative time, postoperative hospital stay, postoperative increase in serum creatinine levels, complication rate, and stone-free rate (SFR) at 1 week and 4 weeks postoperatively were compared among the three groups. Linear regression was employed to identify independent predictors of operative time, while logistic regression was used to analyze independent factors influencing the stone-free rate at 4 weeks postoperatively and the occurrence of complications.

**Results:**

All patients in the three groups successfully completed the surgery. The operative time in group A was (61.5 ± 8.9) min, which was significantly longer than that in group B [(53.4 ± 6.3) min] and group C [(51.0 ± 6.7) min] (*P* < 0.01). There were no significant differences in the postoperative hospital stay among group A [(2.2 ± 0.8) d], group B [(2.2 ± 0.7) d], and group C [(2.1 ± 0.3) d] (*P*>0.05). No significant differences were observed in the postoperative increase in serum creatinine levels among group A [(12.23 ± 8.68) μmol/L], Group B [(13.34 ± 9.11) μmol/L], and group C [(12.16 ± 8.38) μmol/L] (*P* > 0.05). The complication rate was 7.1% in group A, with no significant differences compared to 8.1% in group B and 8.0% in group C (*P* > 0.05). At 1 week postoperatively, the SFR was 64.2% in group A, with no significant differences compared to 62.2% in group B and 66.0% in group C (*P*>0.05); at 4 weeks postoperatively, the SFR was 85.7% in group A, with no significant differences compared to 89.2% in group B and 90.0% in group C (*P >*0.05). Multivariate logistic regression analysis demonstrated that a history of urinary stone surgery was not an independent influencing factor for the SFR at 4 weeks postoperatively or the occurrence of complications (P>0.05).

**Conclusion:**

A history of PCNL may prolong the operative time of one-stage RIRS, but it has no significant impact on the SFR or complication rate. A history of RIRS has no significant effect on the operative time, stone-free rate, or postoperative complication rate of one-stage RIRS. For patients with renal stones who have previously undergone PCNL or RIRS, one-stage RIRS represents an effective and safe treatment option.

## Introduction

The global incidence of urinary stones is showing an increasing trend year by year. In China, the overall incidence is approximately 6.4%, with a notably higher rate of 13% in southern regions due to climatic and dietary characteristics (1, 2). Among these, renal stones are the most prevalent, and their pathogenesis is closely associated with metabolic abnormalities, urinary tract obstruction, and environmental factors (3, 4). Currently, percutaneous nephrolithotomy (PCNL) and retrograde intrarenal surgery (RIRS) are the one-stage surgical approaches for renal stones. Previously, RIRS was considered to have limited efficacy in treating renal stones with a diameter > 2 cm. However, with the development of technologies such as disposable flexible ureteroscopes and flexible negative-pressure sheaths, the application of RIRS for large renal stones has become increasingly widespread, and one-stage lithotripsy procedures have gradually been promoted in clinical practice (5). Previous surgeries may significantly impact the technical difficulty and perioperative risks of subsequent procedures by altering the intra-renal anatomical structure or the conditions of the operative access (6, 7). Therefore, this study retrospectively compared the efficacy of one-stage RIRS in patients with renal stones who had a history of PCNL or RIRS in our department from January 2022 to June 2025. The aim was to investigate the influence of these two surgical histories on the efficacy and safety of RIRS, providing evidence for clinical decision-making.

## Materials and methods

### Research subjects

A retrospective collection of data was conducted on 115 patients with renal stones who underwent one-stage RIRS in the Department of Urology, Songjiang Hospital Affiliated to Shanghai Jiao Tong University School of Medicine from January 2022 to June 2025. The collected data included general patient information, the Resorlu-Unsal stone score (RUSS) (8), and stone burden. Based on the history of renal stone surgery, the patients were categorized into three groups: Group A (28 patients with a history of ipsilateral PCNL), Group B (37 patients with a history of ipsilateral RIRS), and Group C (50 patients with no history of urinary stone surgery).

Inclusion criteria: (1) Patients with a definitive diagnosis of unilateral renal stones established via CT examination. (2) For single stones, the maximum diameter ≤ 3 cm; for multiple stones, the sum of the maximum diameters ≤ 3 cm. (3) Patients with a history of only ipsilateral PCNL or RIRS in the past. (4) After communication with the patient, the patient is willing to undergo RIRS.

Exclusion criteria: (1) Patients with uncontrolled urinary tract infections. (2) Patients with severe urethral or ureteral strictures, or urinary tract malformations. (3) Patients with comorbid diabetes. (4) Patients taking immunosuppressants or those with immunodeficiency. (5) Patients with a previous history of extracorporeal shock wave lithotripsy (ESWL). (6) Patients who are unable to tolerate surgery due to other reasons.

### Surgical method

After general anesthesia, the patient was placed in the lithotomy position. Routine disinfection and draping were performed. A rigid ureteroscope was inserted through the urethra into the bladder to locate the ureteral orifice on the affected side. After placing a guidewire, a matching flexible negative-pressure ureteroscope sheath (Model: FY-1240B/1150B, Hunan Evide Medical Devices Co., Ltd.) was inserted along the guidewire according to the ureteral conditions, and it was connected to a central negative-pressure device.

Once the sheath was successfully placed, an F8.5 disposable flexible ureteroscope (Model: EU-316710R, Hunan Evide Medical Devices Co., Ltd.) was introduced. The energy and frequency parameters of the holmium laser were adjusted according to the characteristics of the stones, and lithotripsy was performed. After lithotripsy, larger stone fragments were retrieved using a stone basket and removed from the body. After careful examination to confirm the absence of any significant residual stones, the ureteroscope and the flexible sheath were withdrawn. An F6 ureteral double-J stent was placed, and a urinary catheter was inserted.

All procedures in this study were performed by two senior urologists, each with over five years of experience in RIRS surgery.

### Data collection

Operative time (defined as the duration from the insertion of the endoscope into the body until its final withdrawal), complications [determined using the Clavien-Dindo classification system (9)], postoperative hospital stay (discharge criteria: stable vital signs), and stone-free rate (SFR) at 1 week and 4 weeks postoperatively (stone clearance was considered when CT scans showed no residual stones or residual stones with a diameter ≤ 4 mm, accompanied by the absence of clinical symptoms). The indicator for postoperative renal function evaluation was the change in serum creatinine levels on the first postoperative day compared with the preoperative levels.

### Statistical analysis

Data analysis was conducted using SPSS 26.0. Measurement data that followed a normal distribution were presented as mean ± standard deviation. For comparisons among multiple groups, one-way analysis of variance (ANOVA) was applied, and pairwise comparisons between groups were performed using the LSD-t test. Categorical data were analyzed with the chi-square test. Linear regression analysis was employed to identify the independent predictors of operative time, and logistic regression analysis was utilized to determine the independent influencing factors of the SFR at 4 weeks postoperatively and the occurrence of complications. A P-value of less than 0.05 was considered to indicate a statistically significant difference.

## Result

### General information and renal stone parameters of the three groups of patients

Patients in the three groups showed no significant differences in terms of age, gender, BMI, as well as stone location, size, and CT values (*P*>0.05), as shown in **Table 1**.

**Table 1:** Comparison of general demographic data and renal stone conditions among the three patient groups.

Indicator	Group A (n=28)	Group B (n=37)	Group C (n=50)	F/X² Value	P Value
Age (years)	48.14 ± 16.81	47.30 ± 14.17	50.10 ± 15.14	0.385	0.681
BMI (kg/m^2^)	21.15 ± 3.86	20.73 ± 4.19	21.13 ± 3.85	4.48	0.47
Gender				0.058	0.971
Male	17 (60.7%)	22 (59.5%)	31 (62.0%)		
Female	11 (39.3%)	15 (40.5%)	19 (38.0%)		
Stone side				0.033	0.984
Left	15 (53.6%)	19 (51.4%)	26 (52.0%)		
Right	13 (46.4%)	18 (48.6%)	24 (58.0%)		
Location of Stone in Renal				0.726	0.994
Upper Calyx	3 (10.7%)	3 (8.1%)	4 (8.0%)		
Middle Calyx	5 (17.9%)	6 (16.3%)	7 (14.0%)		
Lower Calyx	9 (32.1%)	12 (32.4%)	15 (30.0%)		
Renal Pelvis	11 (39.3%)	16 (43.2%)	24 (48.0%)		
Stone Diameter (mm)	20.21 ± 5.05	22.86 ± 5.14	21.78 ± 5.32	2.076	0.130
Stone Surface Area (mm²)	322.81 ± 126.54	332.76 ± 110.41	331.52 ± 113.49	0.069	0.934
Mean Stone CT Value (HU)	945.39 ± 274.06	926.68 ± 263.54	989.40 ± 284.22	0.594	0.554
RUSS score	2.32 ± 1.12	2.46 ± 1.41	2.60 ± 1.08	0.492	0.613

### Operative conditions of the three groups of patients

All patients successfully underwent the operation. The operation time in group A was significantly longer than that in group B and group C (*P* < 0.01), while there was no significant difference in operation time between Groups B and C. No acute renal injury occurred in any of the three groups. There were no significant differences among the three groups in terms of postoperative hospital stay, postoperative changes in serum creatinine, SFR at 1 week after surgery, and SFR at 4 weeks after surgery (P>0.05), as shown in **Table 2**.

**Table 2:** Comparison of surgical conditions among the three patient groups.

Indicator	Group A (n=28)	Group B (n=37)	Group C (n=50)	F/X² Value	P Value
Operation time (min)	61.50 ± 8.91	53.43 ± 6.34	50.96 ± 6.71	19.689	<0.01
Postoperative hospital stays (d)	2.21 ± 0.78	2.19 ± 0.74	2.08 ± 0.27	0.541	0.655
Postoperative increase in serum creatinine (μmol/L)	12.23 ± 8.68	13.34 ± 9.11	12.16 ± 8.38	0.223	0.799
SFR at 1 week postoperatively	64.2% (17/28)	62.2% (23/37)	66.0% (33/50)	0.225	0.894
SFR at 4 weeks postoperatively	85.7% (24/28)	89.2% (33/37)	90.0 (45/50)	0.342	0.843

### Results of linear regression analysis for operative time

The results demonstrated that a history of previous PCNL (t=2.000, *P* = 0.048), stone diameter (t=3.236, *P* = 0.002), stone CT value (t=2.786, *P* = 0.006), and RUSS score (t=2.060, *P* = 0.042) were independent predictors of prolonged operative time. No other indices exerted an independent effect on operative time (*P*>0.05).

### Complication status of the three groups of patients

Both group A and group B had one case each of Clavien-Dindo grade II complications (both cases were urinary tract infection with fever). Regarding Clavien-Dindo grade I complications, there was 1 case in Group A, 2 cases in group B, and 4 cases in group C. All 7 cases of Clavien-Dindo grade I complications showed improved symptoms without special treatment, while the 2 cases of Clavien-Dindo grade II complications improved after antibiotic escalation, as shown in **Table 3**.

**Table 3:** Comparison of postoperative complications among the three patient groups.

Indicator	Group A (n=28)	Group B (n=37)	Group C (n=50)	X² Value	P Value
Postoperative complication (n, %)	5 (7.25%)	9 (7.96%)	8 (7.27%)	0.024	0.988
Clavien-Dindo grade I	4	7	8		
Clavien-Dindo grade II	1	2			

### Results of logistic regression analysis for SFR at 4 weeks postoperatively and complications

Logistic regression analysis demonstrated that after adjusting for relevant confounding factors, stone diameter (OR = 0.807, *P* = 0.011) and stone CT value (OR = 0.996, *P* = 0.046) were independent influencing factors for the SFR at 4 weeks postoperatively. However, a history of previous PCNL or RIRS was not an independent influencing factor for either the postoperative SFR or the complication rate (all *P*>0.05).

## Discussion

With the continuous advancement of endoscopic techniques and lithotripsy equipment, the treatment of renal stones has entered the era of minimally invasive therapy. PCNL and RIRS have become the mainstream minimally invasive surgical procedures, with their effectiveness and practicality widely recognized by urologists. These two surgical approaches each have their own advantages and limitations (10): PCNL offers relatively high lithotripsy efficiency and is particularly suitable for large-volume and complex stones; however, it carries risks of bleeding and potential renal injury, along with a longer postoperative recovery period. RIRS, utilizing natural orifices, results in less trauma, milder pain, and faster recovery. Nevertheless, it faces challenges in terms of lithotripsy efficiency and infection control, especially for stones with high hardness or large volume, which may necessitate longer operative times and multiple treatments to achieve satisfactory lithotripsy outcomes (11, 12). With the progress of technology and the maturation of surgical techniques, particularly the widespread use of flexible negative-pressure sheaths and disposable flexible ureteroscopes, the scope of application for RIRS has been significantly expanded, demonstrating remarkable efficacy, especially in the management of renal stones with diameters exceeding 2 cm and in performing one-stage RIRS (13, 14). However, the successful implementation and outcomes of one-stage RIRS are influenced by a variety of complex factors (6, 7, 15), among which a patient’s history of renal stone surgery is an important factor that cannot be overlooked.

This study conducted a retrospective analysis to compare the impact of different renal calculus surgical histories on the outcomes of one-stage RIR. The operative duration in Group A was significantly prolonged compared to Groups B and C (*P* < 0.05), consistent with findings from previous studies (7, 16). The linear regression analysis in the present study further confirmed that, after adjustment for confounding factors, a history of previous PCNL was an independent predictor of prolonged operative time (P<0.05). This may be attributed to anatomical alterations in the renal collecting system caused by PCNL, such as scarring or stenosis at the calyceal neck, which complicate access to the renal calyces and stone localization during RIRS (17). Additionally, post-PCNL renal parenchymal fibrosis or calyceal morphological changes may further prolong operative time and reduce lithotripsy efficiency in RIRS (18). However, our study demonstrated that a history of prior RIRS did not prolong surgical duration, contrasting with some reports suggesting that post-RIRS infections or ureteral mucosal injuries may increase subsequent procedural difficulty (19). This discrepancy may result from the utilization of negative-pressure suction sheaths, which maintain a low-pressure, clear surgical field and thereby reduce operative time (13). Consequently, for patients with a history of PCNL undergoing one-stage RIRS, surgeons must exercise heightened caution and patience to ensure procedural success and safety.

SFR is one of the crucial indicators for evaluating the postoperative efficacy of urinary stone treatment. The results of this study demonstrate that there is no significant difference in the postoperative SFR among the three groups of patients. Logistic regression analysis further confirmed that previous surgical history had no independent effect on the SFR at 4 weeks postoperatively (*P*>0.05). Alkand et al. (7) reported that for patients who had previously undergone open renal stone surgery, the SFR significantly decreased when they subsequently received RIRS. However, for patients with a history of PCNL, the SFR was not significantly affected when they underwent RIRS again (16). The possible reasons are as follows: Firstly, inherent differences exist in the minimally invasive properties of the surgical procedures themselves. Open surgery, for instance, tends to induce extensive perirenal fibrosis and severe anatomical alterations, which significantly increases the difficulty of subsequent endoscopic interventions. In contrast, PCNL and RIRS, as minimally invasive approaches, result in relatively limited damage to intrarenal structures and exert a relatively minor influence on subsequent RIRS procedures. Secondly, the application of a flexible negative-pressure ureteroscopic sheath extends the operational range of flexible ureteroscopy. Its active suction function can effectively remove intraoperative stone fragments, thereby enhancing the immediate stone-free rate (13, 14) and mitigating the potential impact of a prior surgical history on the SFR.Surgical complications are one of the critical indicators for evaluating the safety and feasibility of a surgical approach. Previous studies have yielded controversial conclusions regarding the impact of a history of PCNL on the complications of subsequent RIRS (16, 20). In the present study, logistic regression analysis confirmed that a history of previous surgery was not an independent influencing factor for the occurrence of complications (P>0.05). Furthermore, this study demonstrated no significant difference in the elevation of serum creatinine levels among the three groups, and no acute kidney injury occurred in any group, suggesting that a history of previous surgery did not increase the risk of renal injury. The results of this study demonstrate that there is no significant difference in the postoperative complication rates among the three groups of patients, and no marked difference is observed in the postoperative changes in serum creatinine levels either. This may be related to factors such as the relatively short surgical duration and the relatively mature surgical techniques employed in this study. Notably, Clavien-Dindo grade II complications occurred in Groups A and B, while only Clavien-Dindo grade I complications were observed in Group C. This discrepancy may be associated with an increased risk of urinary tract infection (UTI) due to the postoperative indwelling of a double-J stent. The double-J stent alters the normal urinary flow pathway, leading to urine stagnation, which provides a breeding ground for bacterial proliferation. Additionally, as a foreign body in the body, the double-J stent has a surface prone to biofilm formation, creating conditions conducive to bacterial aggregation (21, 22). It is evident that although a prior history of PCNL/RIRS may not directly increase the incidence of surgical complications, once complications occur, the consequences may be more severe. This serves as a reminder that surgeons should pay close attention to the impact of a history of PCNL/RIRS on the safety of one-stage RIRS for patients with renal stones.

This study still has some limitations. Firstly, as a single-center retrospective study with a relatively small sample size, this study may be subject to certain selection bias and information bias. Secondly, some novel surgical techniques for urinary calculi were not included, which may have an impact on the study results. Thirdly, this study did not analyze the stratified effect of the interval between surgeries on the therapeutic efficacy, and further subgroup analysis can be conducted in the future to clarify the role of this factor. In the future, it is necessary to conduct further multi-center, large-scale, and prospective studies to comprehensively evaluate the influence of different prior surgical approaches on the outcomes of one-stage RIRS in patients with renal stones.

In summary, a history of PCNL may prolong the operative time of one-stage RIRS in patients with renal stones, but it has no significant impact on the postoperative SFR and complications. Similarly, a history of RIRS does not significantly affect the operative time, postoperative SFR, or complications of one-stage RIRS in renal stone patients. However, this does not imply that clinicians can overlook the influence of prior surgical history on RIRS. In clinical practice, when performing reoperative renal stone surgery, it is essential to fully consider the patient’s history of renal stone surgeries, stone characteristics, and surgical options to develop a personalized treatment plan, thereby ensuring the success and safety of the procedure.

## Conclusion

This study found that a history of PCNL may prolong operative time but does not significantly impact the SFR and complication rates of one-stage RIRS for renal stones. A history of RIRS has no significant effect on these outcomes. One-stage RIRS remains effective and safe regardless of prior surgical history. Personalized treatment planning is essential.

## Data Availability

The raw data supporting the conclusions of this article will be made available by the authors, without undue reservation.
